# Supplementary material for: EnzML: multi-label prediction of enzyme classes using InterPro signatures
Source: BMC Bioinformatics. 2012 Apr 25;13:61. doi: 10.1186/1471-2105-13-61 (PMC3483700; doi:10.1186/1471-2105-13-61)
Supplement: Addtional file 5 — The Java code to format the data files, evaluate and predict. The file enzml_java_code.tar.gz contains the Java code used to format database data to ARFF and XML formats, to execute cross and train-test (jackknife) evaluations and to record evaluation results to database. More information is included in the readme.txt file and the Javadoc files. The code can be used with a MySQL database. To use a different database software, other JDBC drivers might be required. [file 1471-2105-13-61-S5.gz › java_code/utils/doc/uk/ac/ed/inf/utils/tests/UtilsTest.html]

UtilsTest


---


|  |  |  |
| --- | --- | --- |
| **Overview** \| **Package** \| **Class** \| **Use** \| **Tree** \| **Deprecated** \| **Index** \| **Help** | |  |
| **PREV CLASS**   **NEXT CLASS** | **FRAMES**    **NO FRAMES**     **All Classes** |  |
| SUMMARY: NESTED \| FIELD \| CONSTR \| METHOD | DETAIL: FIELD \| CONSTR \| METHOD |  |


---


## uk.ac.ed.inf.utils.tests Class UtilsTest

```
java.lang.Object
  junit.framework.Assert
      junit.framework.TestCase
          uk.ac.ed.inf.utils.tests.UtilsTest
```

**All Implemented Interfaces:**: junit.framework.Test

---

``` public class UtilsTest extends junit.framework.TestCase ```

Class

**Version:**
:   9 Jul 2008

**Author:**
:   Luna De Ferrari luna.deferrari-at-ed.ac.uk

---

**Constructor Summary**

`UtilsTest()`


| **Method Summary** | |
| --- | --- |
| `void` | `testClass()`             public void testBigDecimal(){ this.testBigDecimalGeneric(0.0, 1.0, 0.1); this.testBigDecimalGeneric(0.0, 1.0, 0.2); this.testBigDecimalGeneric(0.0, 1.0, 0.3); } public void testDoubleIncrements1(){ this.testDoubleIncrementsBigDecimal(0.0, 10.0, 0.1); this.testDoubleIncrementsBigDecimal(0.0, 1.0, 0.1); this.testDoubleIncrementsBigDecimal(0.0, 1.0, 0.2); this.testDoubleIncrementsBigDecimal(0.0, 1.0, 0.3); // is not well rounded this.testDoubleIncrementsBigDecimal(0.0, 1.7, 0.3); // increment is null Vector increments = this.testDoubleIncrementsBigDecimal(0.0, 10.0, 0.0); //assertNull(increments); } public Vector testDoubleIncrementsBigDecimal(Double min, Double max, Double percOfIncrement){ TreeSet basicIncrements = Utils.getDoubleIncrementsBigDecimal(min, max, percOfIncrement); if(basicIncrements != null){ Vector increments = new Vector(basicIncrements); Double range = max - min; Double increment = range \* percOfIncrement; if(increment > 0 \|\| increment < 0){ System.out.println("min: " + min + ", max: " + max + ", increment: " + increment + " " + increments.toString()); Double noOfincrements = (range / increment) + 1; int numberOfIncrements = noOfincrements.intValue(); //assertEquals(numberOfIncrements, increments.size()); for(int i=0; i < numberOfIncrements; i++ ){ Double value = min + (i \* increment); //assertEquals(value, (Double) increments.get(i)); } }else{ //assertEquals(null, increments); } return increments; }else{ return null; } } public void testBigDecimalGeneric(Double minDouble, Double maxDouble, Double percentOfIncrementDouble){ BigDecimal min = BigDecimal.valueOf(minDouble); BigDecimal max = BigDecimal.valueOf(maxDouble); BigDecimal percentOfIncrement = BigDecimal.valueOf(percentOfIncrementDouble); Vector intervals = new Vector(); BigDecimal range = max.subtract(min); System.out.println("UtilsTest BigDecimal: " + min + " " + max + " " + percentOfIncrement + " " + range); System.out.println("zero " + BigDecimal.ZERO); assertEquals(1, range.compareTo(BigDecimal.ZERO)); BigDecimal increment = range.multiply(percentOfIncrement); System.out.println("range.divide(increment, RoundingMode.DOWN) " + range.divide(increment, RoundingMode.DOWN)); BigDecimal value = min; value = value.add(increment); System.out.println("value.add(increment) " + value); System.out.println("value.add(increment).ROUND\_DOWN " + value.add(increment).ROUND\_DOWN); System.out.println("max.ROUND\_DOWN " + max.ROUND\_DOWN); System.out.println("---------------------------------"); BigDecimal one0 = BigDecimal.valueOf(1.0); BigDecimal one1 = BigDecimal.valueOf(1.0); BigDecimal range0 = one0.subtract(one1); assertTrue(range0.compareTo(BigDecimal.ZERO) == 0); } |
| `void` | `testDoubleIncrements2()` |
| `void` | `testIncrementsUtil()` |
| `void` | `testIntegerIncrements(java.lang.Integer min, java.lang.Integer max, java.lang.Double incrementAsPercentageOfMaxValue)` |
| `void` | `testObjectIncrements()` |
| `void` | `testSeries()` |

**Methods inherited from class junit.framework.TestCase**

`countTestCases, getName, run, run, runBare, setName, toString`

**Methods inherited from class junit.framework.Assert**

`assertEquals, assertEquals, assertEquals, assertEquals, assertEquals, assertEquals, assertEquals, assertEquals, assertEquals, assertEquals, assertEquals, assertEquals, assertEquals, assertEquals, assertEquals, assertEquals, assertEquals, assertEquals, assertEquals, assertEquals, assertFalse, assertFalse, assertNotNull, assertNotNull, assertNotSame, assertNotSame, assertNull, assertNull, assertSame, assertSame, assertTrue, assertTrue, fail, fail, failNotEquals, failNotSame, failSame, format`

**Methods inherited from class java.lang.Object**

`equals, getClass, hashCode, notify, notifyAll, wait, wait, wait`

**Constructor Detail**

### UtilsTest

```
public UtilsTest()
```


**Method Detail**

### testClass

```
public void testClass()
```

:   public void testBigDecimal(){ this.testBigDecimalGeneric(0.0, 1.0, 0.1);
    this.testBigDecimalGeneric(0.0, 1.0, 0.2);
    this.testBigDecimalGeneric(0.0, 1.0, 0.3); }
    public void testDoubleIncrements1(){
    this.testDoubleIncrementsBigDecimal(0.0, 10.0, 0.1);
    this.testDoubleIncrementsBigDecimal(0.0, 1.0, 0.1);
    this.testDoubleIncrementsBigDecimal(0.0, 1.0, 0.2);
    this.testDoubleIncrementsBigDecimal(0.0, 1.0, 0.3);
    // is not well rounded this.testDoubleIncrementsBigDecimal(0.0, 1.7,
    0.3);
    // increment is null Vector increments =
    this.testDoubleIncrementsBigDecimal(0.0, 10.0, 0.0);
    //assertNull(increments); }
    public Vector testDoubleIncrementsBigDecimal(Double min,
    Double max, Double percOfIncrement){ TreeSet basicIncrements
    = Utils.getDoubleIncrementsBigDecimal(min, max, percOfIncrement);
    if(basicIncrements != null){ Vector increments = new
    Vector(basicIncrements); Double range = max - min; Double
    increment = range \* percOfIncrement;
    if(increment > 0 || increment < 0){ System.out.println("min: " + min +
    ", max: " + max + ", increment: " + increment + " " +
    increments.toString());
    Double noOfincrements = (range / increment) + 1; int numberOfIncrements =
    noOfincrements.intValue();
    //assertEquals(numberOfIncrements, increments.size()); for(int i=0; i <
    numberOfIncrements; i++ ){ Double value = min + (i \* increment);
    //assertEquals(value, (Double) increments.get(i)); }
    }else{ //assertEquals(null, increments); } return increments; }else{
    return null; } }
    public void testBigDecimalGeneric(Double minDouble, Double maxDouble,
    Double percentOfIncrementDouble){ BigDecimal min =
    BigDecimal.valueOf(minDouble); BigDecimal max =
    BigDecimal.valueOf(maxDouble); BigDecimal percentOfIncrement =
    BigDecimal.valueOf(percentOfIncrementDouble);
    Vector intervals = new Vector(); BigDecimal range
    = max.subtract(min);
    System.out.println("UtilsTest BigDecimal: " + min + " " + max + " " +
    percentOfIncrement + " " + range); System.out.println("zero " +
    BigDecimal.ZERO); assertEquals(1, range.compareTo(BigDecimal.ZERO));
    BigDecimal increment = range.multiply(percentOfIncrement);
    System.out.println("range.divide(increment, RoundingMode.DOWN) " +
    range.divide(increment, RoundingMode.DOWN)); BigDecimal value = min;
    value = value.add(increment); System.out.println("value.add(increment) "
    + value); System.out.println("value.add(increment).ROUND\_DOWN " +
    value.add(increment).ROUND\_DOWN); System.out.println("max.ROUND\_DOWN " +
    max.ROUND\_DOWN); System.out.println("---------------------------------");
    BigDecimal one0 = BigDecimal.valueOf(1.0); BigDecimal one1 =
    BigDecimal.valueOf(1.0); BigDecimal range0 = one0.subtract(one1);
    assertTrue(range0.compareTo(BigDecimal.ZERO) == 0);
    }

---


### testDoubleIncrements2

```
public void testDoubleIncrements2()
```

---


### testIncrementsUtil

```
public void testIncrementsUtil()
```

---


### testIntegerIncrements

```
public void testIntegerIncrements(java.lang.Integer min,
                                  java.lang.Integer max,
                                  java.lang.Double incrementAsPercentageOfMaxValue)
```

---


### testObjectIncrements

```
public void testObjectIncrements()
```

---


### testSeries

```
public void testSeries()
```


---


|  |  |  |
| --- | --- | --- |
| **Overview** \| **Package** \| **Class** \| **Use** \| **Tree** \| **Deprecated** \| **Index** \| **Help** | |  |
| **PREV CLASS**   **NEXT CLASS** | **FRAMES**    **NO FRAMES**     **All Classes** |  |
| SUMMARY: NESTED \| FIELD \| CONSTR \| METHOD | DETAIL: FIELD \| CONSTR \| METHOD |  |


---
